# Guidelines for Neuroprognostication in Critically Ill Adults with Status Epilepticus

**DOI:** 10.1007/s12028-025-02425-8

**Published:** 2026-01-08

**Authors:** Dominik Madzar, Venkatakrishna Rajajee, Susanne Muehlschlegel, Katja E. Wartenberg, Sheila A. Alexander, Katharina M. Busl, Claire J. Creutzfeldt, Gabriel V. Fontaine, David Y. Hwang, Keri S. Kim, Dea Mahanes, Shraddha Mainali, Juergen Meixensberger, Oliver W. Sakowitz, Panayiotis N. Varelas, Christian Weimar, Thomas Westermaier, Sara E. Hocker

**Affiliations:** 1 Department of Neurology, Friedrich-Alexander-Universität Erlangen-Nürnberg, Erlangen, Germany; 2 Departments of Neurology and Neurosurgery, University of Michigan, Ann Arbor, MI USA; 3 Departments of Neurology and Anesthesiology/Critical Care Medicine, Division of Neurosciences Critical Care, Johns Hopkins University School of Medicine, Baltimore, MD USA; 4 Department of Neurology, University of Leipzig, Leipzig, Germany; 5 School of Nursing, University of Pittsburgh, Pittsburgh, PA USA; 6 Departments of Neurology and Neurosurgery, College of Medicine, University of Florida, Gainesville, FL USA; 7 Department of Neurology, University of Washington, Seattle, WA USA; 8 Departments of Pharmacy and Neurosciences, Intermountain Health, Salt Lake City, UT USA; 9 Department of Neurology, University of North Carolina, Chapel Hill, NC USA; 10 Department of Pharmacy Practice, University of Illinois at Chicago, Chicago, IL USA; 11 Departments of Neurology and Neurosurgery, UVA Health, Charlottesville, VA USA; 12 Department of Neurology, Virginia Commonwealth University, Richmond, VA USA; 13 Department of Neurosurgery, University of Leipzig, Leipzig, Germany; 14 Department of Neurosurgery, Neurosurgery Center Ludwigsburg-Heilbronn, Ludwigsburg, Germany; 15 Department of Neurology, Albany Medical College, Albany, NY USA; 16 Institute of Medical Informatics, Biometry and Epidemiology, University Hospital Essen, Essen and BDH-Cliniclinic Elzach, Elzach, Germany; 17 Department of Neurosurgery, Helios Amper-Klinikum Dachau, Dachau, Germany; 18 Department of Neurology, Saint Luke’s Health System, Kansas City, MO USA

**Keywords:** Status epilepticus, Functional outcome, Mortality, Prognosis

## Abstract

**Background:**

Status epilepticus (SE) is a heterogeneous disorder with significant morbidity and mortality. This guideline provides broad principles of neuroprognostication and recommendations on the reliability of clinical predictors of outcome that clinicians may consider when counseling surrogate decision-makers of patients with SE.

**Methods:**

This narrative systematic review used Grading of Recommendations Assessment, Development and Evaluation methodology. Good practice recommendations addressed essential principles of neuroprognostication. Candidate predictors, including clinical variables and prediction models, were selected based on clinical relevance and the availability of appropriate evidence. The question was: “When counseling surrogates of patients with SE, should [predictor, with time of assessment if appropriate] be considered a reliable predictor of [outcome] assessed at [time point]?” Outcomes were selected and rated by the panel. Screening criteria were used to exclude smaller and lower-quality studies. Following construction of the evidence profile and summary of findings, recommendations were based on four Grading of Recommendations Assessment, Development and Evaluation criteria: quality of evidence, balance of desirable and undesirable consequences, values and preferences, and resource use.

**Results:**

Good practice recommendations include establishing appropriate long-term goals with surrogates of patients with SE that may extend beyond seizure control alone, setting expectations for recovery in patients with refractory/super-refractory SE, using predictors specific to underlying pathologies as a basis for neuroprognostication, considering potential confounders, and deferring neuroprognostication in cases of unclear etiology until appropriate diagnostic evaluation is performed. Nine clinical variables and two prediction models were selected. A sufficient body of evidence was available only for the prediction of mortality. Forty-two articles met the eligibility criteria for guiding recommendations. None of the variables and models selected were identified as reliable predictors of mortality in patients with SE.

**Conclusions:**

This guideline provides broad principles for neuroprognostication and recommendations on the reliability of predictors of in-hospital mortality in the context of counseling surrogates of patients with SE.

**Supplementary Information:**

The online version contains supplementary material available at 10.1007/s12028-025-02425-8.

## Introduction

Status epilepticus (SE) is a prolonged seizure in which the brain has exhausted the inhibitory mechanisms that result in the natural cessation of the majority of seizures. The most recent definition and classification of SE by the International League Against Epilepsy (ILAE) describes it as “a condition resulting either from the failure of the mechanisms responsible for seizure termination or from the initiation of mechanisms which lead to abnormally prolonged seizures (after time point t1).” It is further defined as “a condition that can have long-term consequences (after time point t2), including neuronal death, neuronal injury, and alteration of neuronal networks…” [1]. The new definition and classification highlight the relationship between seizure type and duration on the development of adverse sequelae.

Four prospective population-based studies have been conducted that included both convulsive and nonconvulsive SE and used a definition incorporating a 30-min minimum duration. These report a minimum incidence of SE of approximately 10 to 15/100,000 in Switzerland [2], Germany [3], and Italy [4], and 60/100,000 in the United States (Richmond, VA) [5]. Although incidence has increased over time, mortality rates have largely remained stable [6, 7]. A study of first episodes of SE showed that over half of cases occur in the absence of known epilepsy [8].

Refractory SE (RSE) is defined as SE that persists after an appropriately selected and dosed benzodiazepine and second antiseizure drug [9, 10], although most studies examining RSE do not strictly follow the “appropriately selected” and “appropriately dosed” elements of this definition. In the only prospective study of RSE at the time of this writing, approximately 23% of patients admitted to the hospital with SE failed to respond to first-line and second-line therapy, wherein first-line therapy was a benzodiazepine and second-line therapy was either phenytoin, valproate, or levetiracetam [10]. Retrospective studies have reported rates of RSE between 31 and 43% [11–13]. “Super-refractory” SE (SRSE) is defined as continuous or recurrent seizures lasting 24 h or more following initiation of anesthetic medications for treatment of SE, including cases in which seizure control is initially achieved but is lost upon weaning the anesthetic drugs [14]. The best estimate of SRSE incidence is from Finland, where, in a study of 15 hospitals providing coverage for a population of 3.9 million, the annual incidence of SRSE was found to be 0.7/100,000 (95% confidence interval [CI] 0.6–0.9) [15]. Between 20 and 41% of people with RSE will progress to become super-refractory [10, 15].

Mortality rates in the literature have varied greatly based on inclusion and exclusion criteria—specifically, age, seizure classification, and etiology. Mortality rates range between 2 and 40% and are lowest when the etiology is fever, intoxication, drug/alcohol withdrawal, trauma, or subtherapeutic antiseizure drug levels from medication withdrawal/noncompliance [11, 13, 16–21]. RSE has a higher mortality compared with nonrefractory SE (~ 20% vs ~ 10%), and the mortality of SRSE is even higher (up to 40%) [10–13, 15] [22]. Concerningly, mortality rates may be higher when evaluated six months to one year after discharge. In the Finnish population-based study referenced previously, hospital mortality was 7.4% (95% CI 0–16.9%), and 1-year mortality was 25.4% (95% CI 21.2–29.8%) [15].

The cause of death in patients hospitalized with SE varies significantly and may be influenced by physician practice and patient population. Withdrawal of life-sustaining therapy (WLST) is the ultimate cause of death in many of these patients [23], and it is likely that in these cases, WLST is preceded by prognostication of poor outcome by a clinician. Thus, it is of critical importance that prognostication be performed accurately on the basis of appropriately validated predictors. Validation of predictors of outcome in patients with SE is made challenging by the variable presentations and complications resulting from the seizures, systemic sequelae, and treatments. Prognostication during counseling is both essential and inevitable, however, and occurs routinely in intensive care units worldwide. The objective of these guidelines from the Neurocritical Care Society (NCS) and Deutsche Gesellschaft für Neurointensivmedizin (DGNI) is to outline broad principles of neuroprognostication in adults who are critically ill with SE, ensure that such prognostication is performed on the basis of the most reliable predictors available, and highlight opportunities for future research.

### Scope, Purpose, and Target Audience

The scope of these Grading of Recommendations Assessment, Development and Evaluation (GRADE) guidelines is the prognostication of neurological outcome in adult patients who are critically ill with SE who receive guideline-concordant, standard-of-care treatment [24, 25]. Notably, these guidelines assume the use of standard-of-care treatment and should not be used to determine the value or intensity of therapeutic measures. The purpose of these guidelines is to provide evidence-based recommendations on the reliability of predictors of neurological outcome in adult patients who are critically ill with SE who have received standard-of-care treatment to aid clinicians in formulating a prognosis. The target audience consists of clinicians responsible for such counseling.

### How to Use these Guidelines

These guidelines provide recommendations on the reliability of select demographic and clinical variables as well as prediction models when counseling families and surrogates of patients with SE who receive standard-of-care treatment. To maintain consistency with published NCS treatment guidelines [24], the panel considered the standard of care to include the use of continuous electroencephalographic (EEG) monitoring when available. Because continuous EEG is resource intensive and may not be widely available, serial intermittent EEG studies were considered an acceptable alternative when continuous monitoring was unavailable. We categorized predictors as reliable, moderately reliable, or not reliable. We based this categorization on a GRADE-based assessment of certainty in the body of evidence as well as effect size (quantification of predictor accuracy) across published studies, as shown in Table 1.

**Table 1:** Reliable and moderately reliable predictors

Category of predictor/model	GRADE criteria					Point estimates of accuracy in the body of evidence	Use during counseling of patients or surrogates?	Presence of additional specific reliable or moderately predictors required for use during counseling?	Suggested language during counseling of patients or surrogates	
	Risk of Bias	Inconsistency	Imprecision	Indirectness	Quality of Evidence- Overall				Likelihood of outcome	Disclaimer of Uncertainty during counseling
Reliable	One downgrade permitted	Downgrade NOT permitted	Downgrade NOT permitted	Downgrade NOT permitted	Moderate or High	High. Prediction models require AUC > 0.8, no evidence of miscalibration in external validation studies	Yes	Preferred, but not absolutely required	“Very likely”	Present, but low
Moderately reliable	One downgrade permitted	Downgrade NOT permitted	One downgrade permitted	One downgrade permitted	Any	High	Yes	Yes	“Likely”	Substantial
Moderately reliable clinical prediction models	One downgrade permitted	Downgrade NOT permitted	One downgrade permitted	One downgrade permitted	Any	High. Prediction models require AUC > 0.7, some miscalibration allowed in external validation studies	Yes	No	Use predicted probability of outcome	“The predicted probability is an estimate, subject to considerable uncertainty”
Not reliable	Downgrade permitted	Downgrade permitted	Downgrade permitted	Downgrade permitted	Any	Any	*No	Not applicable	Not applicable	Not applicable

^*^Many predictors designated “not reliable” are practically utilized by clinicians in formulating and communicating real-world subjective impressions of prognosis. The purpose of these guidelines is to identify predictors, if any, that meet reliable or moderately reliable criteria

A key distinction exists between a reliable predictor of outcome in the context of counseling surrogates of patients requiring life-sustaining therapy and an independent predictor of outcome. An independent predictor fulfills one criterion—a statistically significant association with the outcome of interest in an appropriately conducted multivariate analysis. Independent predictors of outcome may be used for risk stratification, for selection of patients for targeted treatment (such as chemotherapy regimens for cancer), or as building blocks of clinical prediction models. A reliable predictor in the context of counseling the surrogates of patients requiring life-sustaining therapy must be independent but also meet a higher threshold, as described in the “Evidence to recommendation criteria” section. Confidence in the accuracy of the predictor should be sufficiently high to overcome concerns about the undesirable consequence of inappropriate WLST.

Reliable predictors, for the purposes of these guidelines, may be used to formulate a prognosis when the appropriate clinical context is present in the absence of potential confounders. These are predictors with a low rate of error in prediction of poor outcomes, with at least moderate certainty in the body of evidence. When the prognosis is formulated on the basis of one or more reliable predictors, the clinician may describe the outcome as “very likely” during counseling. Given the inherent limitations in neuroprognostication research, the clinician must nevertheless acknowledge the presence of uncertainty—albeit, low—in the prognosis during counseling. Moderately reliable predictors may be used for prognostication only when additional reliable or moderately reliable predictors are present in addition to the appropriate clinical context. These are predictors with a low rate of error in prediction of poor outcomes but with lower certainty in the body of evidence, frequently as a result of smaller studies that result in imprecision. When the prognosis is formulated on the basis of multiple moderately reliable predictors, the clinician may describe the outcome as “likely” during counseling but must acknowledge “substantial” uncertainty in the prognosis. Predictors deemed not reliable should not be used in prognostication. Variables deemed not reliable may be a component of reliable or moderately reliable prediction models.

## Methods

An in-depth description of the methodology used in these guidelines is available in Supplementary Appendix 1.

### Selection of Guideline Questions

Candidate predictors were selected based on clinical relevance and the presence of an appropriate body of literature. Candidate predictors and prediction models were considered “clinically relevant” if, in the subjective opinion of the content experts and guideline chairs, the predictor or components of the prediction models were (1) accessible to clinicians, although universal availability was not required, and (2) likely to be considered by clinicians when counseling patients and surrogates regarding prognosis. Predictors thought particularly likely to be considered by clinicians during prognostication were prioritized. An appropriate body of literature was considered to be present for clinical prediction models with at least 1 external validation study of at least 50 patients in addition to the initial report on development of the model (also with a minimum of 50 patients).

Based on these criteria, the following candidate predictors were selected:

#### Clinical Variables

1. Age
2. Extent of comorbidities
3. Etiology of SE: acute symptomatic or potentially fatal
4. History of seizures/epilepsy
5. Classification of SE according to seizure type/semiology
6. Level of consciousness before treatment
7. Duration of SE
8. Treatment refractoriness: RSE defined as SE that persists after an appropriately selected and dosed benzodiazepine and second anticonvulsant drug
9. Treatment refractoriness: SRSE defined as continuous or recurrent seizures lasting 24 h or more following initiation of anesthetic medications for treatment of SE, including cases in which seizure control is initially achieved but is lost upon weaning the anesthetic drugs

Notably, we did not select specific patterns on EEG other than the persistence (refractoriness) of seizures as prognostic indicators in this systematic review, because they were no longer thought to be widely used by clinicians for this purpose and lacked a sufficient body of evidence. Specific EEG patterns have prognostic value in individual disease states, such as in survivors of cardiac arrest [26]. Studies have suggested that periodic discharges may, in particular, be associated with either a late stage of SE [27] or severity of underlying injury [28, 29]. However, the prognostic value of these patterns across disease states that result in SE independent from etiology and duration of SE could not be meaningfully evaluated in this systematic review.

#### Clinical Prediction Models

1. Status Epilepticus Severity Score (STESS)
2. Modified STESS (mSTESS)
3. Epidemiology-based Mortality score in Status Epilepticus (EMSE)

The population/intervention/comparator/outcome/time frame/setting (PICOTS) question was then framed for the specific candidate predictors as follows: “When counseling family members/surrogates of patients with SE, should [predictor, with time of assessment if appropriate] be considered a reliable predictor of [outcome, with time frame of assessment]?”.

### Selection of Outcomes

The outcomes rated “critical” using the GRADE 1–9 scale were mortality (average rating 9) assessed at or beyond 3 months from hospital discharge, in-hospital mortality (average rating 9) assessed at hospital discharge, functional outcome (average rating 9) assessed at or beyond 3 months from hospital discharge, cognitive outcome (average rating 9) assessed at or beyond 3 months from hospital discharge, and psychiatric or behavioral outcomes (average rating 9) assessed at or beyond 3 months from hospital discharge. The most common time points for assessment of both mortality and functional outcome in the literature were hospital discharge, one month and three months after hospital discharge, last follow-up, and intensive care unit discharge. Functional outcome at hospital discharge was judged by the panel to provide a poor representation of meaningful long-term outcome because progressive improvement after discharge is well documented following SE [30, 31]. No studies that included cognitive, psychiatric, or behavioral outcomes met other full-text screening criteria for the systematic review. The panel was unable to provide recommendations on predictors of functional outcome because there was an insufficient body of evidence meeting our eligibility criteria that assessed this outcome at an appropriate time point. In addition, there was marked inconsistency in the evaluation of functional outcome, which was variably assessed in the literature using modified Rankin Scale (mRS)/Glasgow Outcome Scale cutoffs, comparison of functional status before and after SE based on before versus after mRS/Glasgow Outcome Scale, or poorly characterized criteria, such as “new impairments,””new disabilities,” or “functional decline after SE.” The panel could only provide recommendations on the reliability of predictors of in-hospital mortality, the only outcome with a sufficient body of evidence that met our eligibility criteria. The panel, however, recognized that death following SE frequently occurs as a result of WLST [23] and that self-fulfilling prophecy was an important source of confounding in the body of evidence that addressed this outcome.

### Systematic Review Methodology

**Fig. 1:**
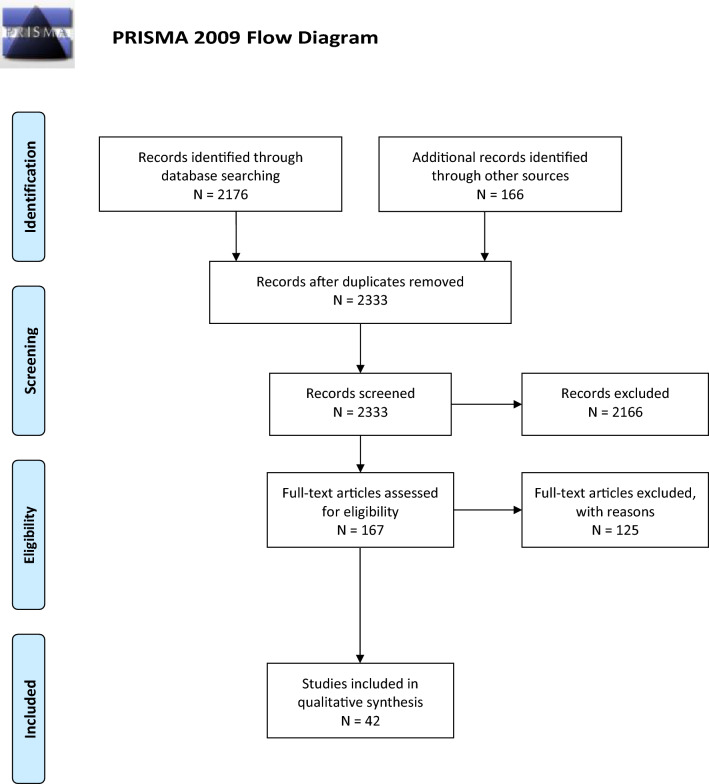
Preferred reporting items for systematic reviews and meta-analyses (PRISMA) flow diagram

An in-depth description of systematic review methodology overall for the NCS-DGNI neuroprognostication guidelines project is in Supplementary Appendix 1, including the librarian search string used for this systematic review. The Preferred Reporting Items for Systematic Reviews and Meta-Analyses flow diagram is in Fig. 1. The initial librarian search was performed February 20, 2019, and encompassed the period of 1946 to the search date. An updated search was performed in March 2022. Full-text screening was performed with the following exclusion criteria: sample size less than 50, studies focused on a highly selected subgroup (such as immune-mediated SE), studies of predictors not established as independent with multivariate analysis, studies focused on a genetic polymorphism as a predictor, and studies of clinical prediction models that did not report model discrimination. Studies of neuroimaging and laboratory biomarkers were not included because of lack of an appropriate body of evidence at the time of full-text screening and lack of widespread use in clinical practice, respectively.

Studies with no restrictions on WLST and likely incorporation of predictors under investigation into clinical neuroprognostication during the course of the study were considered to have a high risk of bias from self-fulfilling prophecy. Studies from countries with restrictions or cultural limitations on WLST were judged to have a lower risk of bias from self-fulfilling prophecy [32].

A summary of individual studies of predictors is in Supplementary Appendix 3. The GRADE evidence profile and summary of findings (for in-hospital mortality are in Table 2.

**Table 2:** GRADE evidence profile/summary of findings table: neuroprognostication- status epilepticus

Outcome	Predictor	Quality of evidence					Summary of Findings (Narrative of effect size)
		RoB	Inconsistency	Indirectness	Imprecision	QoE- Summary (High/ Moderate/ Low/ Very Low)	
*Individual predictors*							
In-hospital mortality	Age	↓	↓			Low	Point estimate of odds ratio for in-hospital mortality 0.98–1.87 (per increasing year)
In-hospital mortality	Extent of comorbidities (as measured by the Charlson Comorbidity Index)	↓				Moderate	Point estimate of odds ratio for in-hospital mortality 1.16–1.31
In-hospital mortality	SE etiology: acute symptomatic or potentially fatal	↓	↓		↓	Very low	Point estimate of odds ratio for in-hospital mortality 0.6–11.7
In-hospital mortality	SE etiology: history of previous seizures/epilepsy	↓	↓		↓	Very Low	Point estimate of odds ratio for in-hospital mortality 0.2–1-4
In-hospital mortality	Seizure type/semiology	↓	↓		↓	Very Low	Point estimate of odds ratio for in-hospital mortality for seizure types generalized consvulsive or NCSE in coma 2.2–4.2; for GCSE and focal motor SE not significant
In-hospital mortality	Level of consciousness before treatment	↓	↓		↓	Very low	Inconsistency in measurement of extent of consciousness impairment (e.g., GCS, coma ±); extremely wide range of odds ratios reported (0.8 – 152)
In-hospital mortality	SE duration	↓	↓		↓	Very low	Point estimate of odds ratio for in-hospital mortality per SE hour 1.001, per day 1.05–1.08
In-hospital mortality	Refractory SE	↓	↓		↓	Very low	Point estimate of odds ratio for in-hospital mortality 0.4–5.3
In-hospital mortality	Super-refractory SE (treatment with anesthetics)	↓	↓		↓	Very low	Point estimate of odds ratio for in-hospital mortality 0.4–9.7
*Prediction models*							
In-hospital mortality	Status epilepticus severity score (STESS)	↓ (Statistical analysis, Study confounding)			↓	Low	AUCs for in-hospital mortality 0.63–0.84; lower boundary of confidence intervals down to ~ 0.5; low PPV 27–33% (cut-off 3) for mortality; uncertainty about best cut-off (3 vs 4 points?)
In-hospital mortality	modified Status epilepticus severity score (mSTESS)	↓ (Statistical analysis, Study confounding)			↓	Low	AUCs for in-hospital mortality 0.62–0.8; lower boundary of confidence intervals down to ~ 0.5; low PPV 20–40% in validation studies
In-hospital mortality	Epidemiology-based mortality score in status epilepticus (EMSE)	↓ (Statistical analysis, Study confounding)				Moderate	AUCs for EMSE-EACE (original version of the score): 0.69 -0.9; optimal cut-off 64/79? Also used in variations: EMSE-EAL/EAC: AUCs 0.65–0.9

GRADE evidence profile and summary of findings. Area Under the Curve (AUC); Epidemiology-based mortality score in status epilepticus (EMSE) which includes the following variants based on variables included: etiology/ age/ comorbidities (EMSE-EAC), etiology/ age/ comorbidities/ electroencephalography-pattern (EMSE-EACE), and etiology/ age/ level of consciousness (EMSE-EAL); Glasgow Coma Scale (GCS); Generalized Convulsive Status Epilepticus (GCSE); Nonconvulsive Status Epilepticus (NCSE); Positive Predictive Value (PPV); Quality of Evidence (QoE); Risk of Bias (RoB); Status Epilepticus (SE); Status epilepticus severity score (STESS)

### Evidence to Recommendation Criteria

#### Quality of Evidence/Certainty in the Evidence and Effect Size

For the purposes of these guidelines, predictors described as “reliable” have both a higher overall certainty in the evidence and greater effect size than “moderately reliable” predictors (Table 1). For “reliable” predictors, one downgrade was permitted for risk of bias, but none were permitted for inconsistency, imprecision, or indirectness, and the overall quality of evidence had to be high or moderate. “Reliable” prediction models were required to demonstrate an area under the receiver operating curve (AUC) of > 0.8. Another important measure is model calibration, or the ability to correctly specify the probability of an outcome. Model calibration is typically reported as a goodness of fit, often using the Hosmer–Lemeshow test or with a calibration curve, slope, or intercept [33]. Assessment of calibration in at least one external validation study of adequate quality per our eligibility criteria was required for reliable prediction models. Single downgrades in each of the domains of risk of bias, imprecision, and indirectness were permitted for “moderately reliable” predictors, but a downgrade for inconsistency was not. In addition, “moderately reliable” prediction models were required to demonstrate an AUC > 0.7, and some miscalibration in some external populations was allowed. Predictors that did not fit “reliable” or “moderately reliable” criteria were classified as “not reliable.”

#### Balance of Desirable and Undesirable Consequences

Accurate prediction of a poor outcome provides family/surrogates with information needed to make decisions aligned with the estimated wishes of the patient. In addition to providing family and surrogates with a greater degree of certainty in the anticipated course of events, it may provide a sense of closure and some degree of comfort from respecting the patient’s wishes. Inaccurate prediction of a poor outcome (i.e., a false-positive prediction of poor outcome) may lead to WLST in an individual who may otherwise have made a meaningful recovery. Because WLST almost always leads to death, the undesirable consequence of an inaccurate prediction of poor outcome was the primary concern during discussions on the reliability of a predictor.

#### Values and Preferences

The panel, including the patient representative, was in agreement that most patients and their surrogates would likely consider an inaccurate prediction of poor outcome that led to the death of a patient who might otherwise have had a reasonable recovery to be far more undesirable than a prolonged period of uncertainty in the outcome. Therefore, a high certainty in the evidence of predictor or prediction model accuracy was necessary to recommend consideration when counseling families and surrogates on prognosis in this context. However, the panel recognized that values and preferences vary greatly and that many consider prolonged dependence of life-sustaining measures, such as artificial enteral nutrition and an artificial airway extending over months or even years, to be unacceptable. This criterion was therefore challenging to apply to frame recommendations applicable to the broad range of patient and surrogate preferences in this disease.

#### Resource Use

The predictors evaluated in our systematic review did not require any expenditure of resources beyond the provision of standard medical care. Similar to other conditions, an accurate prediction of poor outcome in SE may avoid the extended use of resources, over days to years, in patients destined to suffer a poor outcome. The use of resources was therefore thought to favor consideration of a predictor or prediction model during prognostication when confidence in its predictive accuracy was high.

### Good Practice Statements

In accordance with recommendations of the GRADE network, these statements were considered by the panel to be actionable, supported by indirect evidence when appropriate, and essential to guide the practice of neuroprognostication [34]. The good clinical practice reflected in these statements lacked a meaningful body of direct evidence—typically because of insufficient clinical equipoise—but were considered by the panel to be unequivocally beneficial.

#### Good Practice Statement 1

We recommend that clinicians establish with patient surrogates the most appropriate long-term goals of care in individual patients with SE. Such goals, which may range from control of seizures to a return to functional independence, should serve as the basis for prognostication in an individual patient (strong recommendation, evidence cannot be graded).

#### Rationale

In the setting of SE, discussions with surrogates may be centered around or restricted to the prognosis for control of seizures, whereas the primary concern of patients and families may be long-term recovery. Goals of care vary widely, and clarity on acceptable outcomes is critical to prognostication. This must be established with surrogates through active listening and shared decision-making. In adults with the Lennox–Gastaut syndrome and other diseases that cause profound baseline neurological impairment, goals of care may be limited to the control of seizures and a return to home. Prognostication may therefore focus on the probability of termination of SE and the anticipated duration of endotracheal intubation or institutional care. In contrast, surrogates of a patient who is elderly and intubated with a subdural hematoma and SE may prioritize (based on a best estimate of the patient’s wishes) a long-term return to functional independence. The primary focus of prognostication in such a patient should therefore be on the likelihood of functional independence rather than termination of SE or absence of seizures alone.

#### Good Practice Statement 2

Surrogates of patients with RSE/SRSE should be counseled that treatment measures such as prolonged sedative infusions and long-acting antiseizure medications (ASMs) may result in prolonged coma and dependence on life-sustaining measures, such as an artificial airway and artificial enteral nutrition, for a variable duration ranging from days to months. Surrogates should also be counseled that medical complications related to critical illness, hospital-acquired infections, and prolonged restriction of mobility are common in such patients (strong recommendation, evidence cannot be graded).

#### Rationale

In patients with RSE/SRSE, it is critical that expectations for patient recovery be managed appropriately. Surrogates may expect rapid awakening following successful initial control of seizures, whereas neurological improvement may be considerably delayed. Recurrence of seizures on weaning of sedatives may necessitate prolonged infusion use and/or the initiation of ASMs with a prolonged duration of action (such as barbiturates). Patients with RSE and SRSE sometimes require prolonged use of life-sustaining therapy, including tracheostomy and percutaneous gastrostomy [35]. These patients are at risk for a variety of medical complications, such as hospital-acquired infections, venous thromboembolism, aspiration syndrome, and pressure-related injury [35].

#### Good Practice Statement 3

Surrogates of patients with RSE/SRSE should be counseled that a long-term recovery that reaches or approximates the patient’s baseline level of functioning is feasible. Factors such as underlying acute brain injury, comorbidities, nosocomial infections, and sequelae of critical illness may, however, limit functional recovery (strong recommendation, evidence cannot be graded).

#### Rationale

Although patients with RSE/SRSE often require prolonged support, multiple studies indicate the possibility of recovery to long-term functional independence (or the patient’s functional baseline) following weeks or months of coma and life-sustaining treatment [15, 36–38]. Prediction of the duration of dependence on life-sustaining therapy and delayed awakening can be highly challenging. An appropriate period of treatment and observation for delayed recovery in such cases should be established based on a best estimate by surrogates of the patient’s willingness to undergo prolonged life-sustaining care.

#### Good Practice Statement 4

We recommend that in patients with SE and an established underlying etiology, validated predictors of outcome specific to the underlying disease serve as a basis for neuroprognostication (strong recommendation, evidence cannot be graded).

#### Rationale

Although no single etiology of SE is universally associated with a poor prognosis, individual patients may demonstrate reliable predictors of outcome specific to the underlying disease. For example, a patient who is comatose with RSE/SRSE following cardiac arrest may demonstrate a bilateral absence of N20 cortical responses with preservation of extracranial conduction and evidence of diffuse loss of grey–white differentiation on computed tomography of the brain [26]. These reliable or moderately reliable predictors of outcome specific to the underlying condition (anoxic injury) may serve as the basis for prognostication of a poor outcome.

#### Good Practice Statement 5

We recommend that neuroprognostication, when feasible, should be performed in the absence of potential confounders of the neurological examination, such as residual sedation, a toxic-metabolic encephalopathy or prolonged postictal encephalopathy (strong recommendation, evidence cannot be graded).

#### Rationale

Clinicians should avoid premature judgment of the patient’s neurological status without a careful clinical examination in the absence of potential confounders because resolution of the confounder may clarify a good prognosis as the patient awakens. The duration of sedative effect is dependent on factors such as medication half-life, duration of infusion, hepatic and renal function, drug interactions, patient age, temperature, and comorbidities. The involvement of a clinical pharmacist may help clarify the expected duration of sedative effect in complex patients who are critically ill. In addition, SE may occur in patients with conditions such as septic or hepatic encephalopathy [39, 40]. These toxic-metabolic encephalopathies may be entirely reversible but may confound the patient’s neurological assessment for a prolonged duration. Though also reversible, a prolonged postictal encephalopathy may be challenging to identify. Although a typical postictal period persists for minutes to hours, prolonged periods of impaired consciousness of four to ten days have been reported [41]. Risk factors for a prolonged postictal period may include static encephalopathy, longer duration of SE, history of SE, prior prolonged postictal state, diffuse structural brain disease, older age, and baseline impairment of functional status [41–43]. Neuroprognostication should be deferred in these settings for an appropriate period of time that is established based on a careful evaluation of individual patient factors.

Rapid weaning of sedating medication may not be feasible in some patients who are critically ill, such as those with RSE/SRSE, elevated intracranial pressure, or acute respiratory distress syndrome. In these settings, surrogates should be counseled on the likelihood of prolonged uncertainty in the outcome (see good practice statement 2) [15, 35–38].

#### Good Practice Statement 6

We recommend that in patients with SE of unclear etiology neuroprognostication be deferred until an appropriate diagnostic evaluation is performed for the underlying disease process. Evaluation should be timely and, when feasible, be performed concurrently with treatment of seizures, as etiology may determine appropriate treatment and prognosis (strong recommendation, evidence cannot be graded).

#### Rationale

Although no single underlying disease that results in SE confers a universally poor prognosis, the outcome may be critically dependent on timely diagnosis and treatment. For example, mortality drops from about 70% to 28% with the timely initiation of antiviral therapy in herpes simplex virus 1 encephalitis [44]. Patients with new-onset RSE do not have a clear acute or active structural, toxic, or metabolic cause after 72 h, whereas febrile infection-related epilepsy syndrome is a subcategory that requires a prior febrile infection between 2 weeks and 24 h prior to RSE onset [9]. Although a significant proportion of such patients will die (16–27%) or suffer long-term cognitive or functional impairment (50–75%) [45], establishing an etiology such as an autoimmune disease may modify prognosis through the use of appropriate treatment, such as immunomodulatory therapy.

## Recommendations: Clinical Variables as Predictors

### Outcome: in-Hospital Mortality

#### Question 1

When counseling surrogates of patients with SE, should age alone be considered a reliable predictor of in-hospital mortality?

#### Description of the Predictor

Older age generally reflects a higher degree of frailty, more preexisting conditions, and a higher risk of poor outcome after complications of critical care. In most studies of outcome in SE, age is either used as a continuous variable or dichotomized, typically into < 65 years and ≥ 65 years.

#### Recommendation

When counseling surrogates of patients with SE, we suggest that age alone not be considered a reliable predictor of in-hospital mortality (weak recommendation; low-quality evidence).

#### Rationale

The body of evidence was downgraded for risk of bias, with various studies demonstrating potential bias in the Quality In Prognosis Studies (QUIPS) domains of study confounding and statistical analysis. Most studies were associated with a risk of self-fulfilling prophecy. CIs were relatively narrow across studies. Some inconsistency was present. Although the majority of studies showed an association of higher age with mortality [11, 13, 46–50], some did not [38, 51, 52]. It is important to note that there is an age-related likelihood of some SE etiologies, but age does not necessarily determine the severity of these conditions. Older individuals, for instance, may develop SE from a relatively benign cause, such as preexisting poststroke epilepsy, whereas in young individuals without preexisting brain damage, SE may occur as a symptom of a severe acute brain injury (e.g., encephalitis) or the onset of a genetic disease with a poor prognosis (e.g., POLG1 mutations). Therefore, defining an age threshold that accurately predicts the likely outcome is difficult, and age cannot be considered a reliable individual predictor of in-hospital mortality in patients with SE.

#### Question 2

When counseling surrogates of patients with SE, should the extent of comorbidities (as measured by the Charlson Comorbidity Index [CCI]) alone be considered a reliable predictor of in-hospital mortality?

#### Description of the Predictor

Comorbidities can be measured with a variety of scales. The most frequently applied comorbidity measure in SE studies is the CCI, a weighted index designed to predict mortality based on 19 items reflecting the burden of comorbid illness [53]. Higher scores on the CCI were associated with poorer prognosis in various conditions in neurointensive care.

#### Recommendation

When counseling surrogates of patients with SE, we suggest the extent of comorbidities (as measured by the CCI) alone not be considered a reliable predictor of in-hospital mortality (weak recommendation; moderate-quality evidence).

#### Rationale

The body of evidence was downgraded for risk of bias, with various studies demonstrating potential bias in the QUIPS domains of study confounding and statistical analysis. Most studies were associated with a risk of self-fulfilling prophecy. The body of evidence was largely consistent in demonstrating a higher risk of death with higher CCI scores, and CIs were relatively narrow. The CCI was mostly evaluated as a continuous variable, and no potential cutoff value was defined that would be useful for clinicians to predict a good or poor outcome [38, 46, 54–56]. However, the previously mentioned considerations for patient age also apply to the CCI—a higher CCI is not necessarily associated with severe or life-threatening SE etiology.

#### Question 3

When counseling surrogates of patients with SE, should an acute symptomatic or a potentially fatal etiology alone be considered a reliable predictor of in-hospital mortality?

#### Description of the Predictor

SE can develop from various causes. According to ILAE recommendations, etiologies are divided into the categories: (1) acute symptomatic, (2) remote symptomatic, (3) progressive symptomatic, and (4) unknown etiology [1, 57]. Etiologies with a high risk of mortality—whether complicated by SE or not—may be defined as potentially fatal according to a more recent definition [13]. Within this classification, the etiologies considered potentially fatal (independent of the presence of SE) are acute (≤ 7 days) large-vessel ischemic stroke, intracerebral hemorrhage, acute central nervous system (CNS) infection, severe systemic infection, malignant brain tumor, acquired immune deficiency syndrome with CNS complications, chronic renal insufficiency requiring dialysis, systemic vasculitis, metabolic disturbance or acute intoxication sufficient to cause coma in the absence of SE, eclampsia, and intracranial tumor surgery. In contrast, remote or progressive symptomatic conditions, such as previous trauma, stroke, CNS infection, dementia, multiple sclerosis, or meningioma, are considered not potentially fatal.

#### Recommendation

When counseling surrogates of patients with SE, we suggest an acute symptomatic or a potentially fatal etiology alone not be considered a reliable predictor of in-hospital mortality (weak recommendation; very-low-quality evidence).

#### Rationale

The body of evidence was downgraded for risk of bias, with various studies demonstrating potential bias in the QUIPS domains of study confounding, study participation, and statistical analysis. Most studies were associated with a risk of self-fulfilling prophecy. Imprecision and inconsistency were present. Most, but not all, studies reported increased point estimates for mortality in patients with SE due to a potentially fatal etiology [13, 38, 49, 51, 54, 58]. This was mostly true for acute symptomatic causes, but the findings were more uncertain [11, 46, 50, 51, 55, 59, 60]. Acute or potentially fatal etiologies could not be considered reliable, in part because of the problematic definitions. Acute symptomatic causes encompass a wide spectrum of underlying conditions, ranging from severe acute pathologies such as stroke to more innocuous, albeit acute, causes, such as sodium imbalance. Several etiologies cannot be clearly categorized according to ILAE recommendations. Similarly, there is lack of clarity regarding the classification of SE cases without discernible cause as having potentially fatal etiologies. Most importantly, no single diagnosis conveys a universally poor prognosis. Although anoxic injury following cardiac arrest is often considered to be an especially grave etiology of SE [61], there are now several reports of good functional outcome in patients with cardiac arrest who were treated for SE [62, 63] and insufficient evidence to consider SE a reliable indicator of prognosis following cardiac arrest [26].

SE in patients with chronic epilepsy, without acute brain injury or a progressive underlying condition, is often associated with ASM noncompliance or withdrawal, with low levels of ASM in the blood when measured. SE in this setting is associated with a particularly high probability of good long-term outcome, even in the presence of RSE [61]. Surrogates of patients with chronic epilepsy, without acute brain injury or a known progressive underlying etiology, who develop SE/ RSE in the context of possible ASM noncompliance or withdrawal should therefore be counseled that although individual patients may suffer a poor outcome because of comorbidities or complications, the majority of patients will make a good long-term recovery.

#### Question 4

When counseling surrogates of patients with SE, should a history of seizures/epilepsy alone be considered a reliable predictor of in-hospital mortality?

#### Description of the Predictor

SE can occur in individuals with or without a history of seizures/epilepsy. A history of seizures or epilepsy can be determined from patient surrogates, the medical record, or the patient’s regular medications.

#### Recommendation

When counseling surrogates of patients with SE, we suggest a history of previous seizures/epilepsy alone not be considered a reliable predictor of in-hospital mortality (weak recommendation; very-low-quality evidence).

#### Rationale

The body of evidence was downgraded for risk of bias, with various studies demonstrating potential bias in the QUIPS domains of study confounding, study participation, and statistical analysis. Most studies were associated with a risk of self-fulfilling prophecy. Imprecision and inconsistency were present. Although many studies reported lower mortality in patients with SE and a history of seizures/epilepsy, the results were inconclusive [49, 50, 57, 59, 64]. A history of seizures/epilepsy does not necessarily imply a more favorable prognosis, although this may frequently be the case, especially if previous seizures were caused by a disease that itself carries a good prognosis. However, individuals with symptomatic epilepsy due to malignant brain neoplasm or progressive neurodegenerative disease may have a poor long-term prognosis.

#### Question 5

When counseling surrogates of patients with SE, should seizure type/semiology alone be considered a reliable predictor of in-hospital mortality?

#### Description of the Predictor

Semiology refers to the clinical manifestations of seizures, which are variable in SE and range from predominantly motor symptoms, archetypical in tonic–clonic (generalized convulsive) SE, to minor neurologic deficits that may not be identified as the symptoms of SE until EEG evaluation is performed. Similar to epileptic seizures, SE semiology is often categorized based on the prominence of motor symptoms and impairment of consciousness. In 2015, the ILAE proposed a semiological classification for SE [1]. Applying the semiological criteria for single seizures to SE is challenging because seizure type within an SE episode may change with time. Some authors have suggested semiological classification of SE based the “worst seizure type” observed “before initiation of treatment” [13], whereas others emphasize evolution of seizure semiology during an SE episode [65].

#### Recommendation

When counseling surrogates of patients with SE, we suggest seizure type/semiology alone not be considered a reliable predictor of in-hospital mortality (weak recommendation; very-low-quality evidence).

#### Rationale

The body of evidence was downgraded for risk of bias in the QUIPS domains of study confounding and statistical analysis. Inconsistency and imprecision were present. There was often uncertainty about the criteria used for the semiological classification of SE. In the few studies that met eligibility criteria to support recommendations, nonconvulsive SE was associated with in-hospital mortality [47, 49, 51]. Semiology of SE could not be recommended as a predictor because of the very low quality of evidence, with particularly substantial risk of bias from variability in definitions of SE semiology.

#### Question 6

When counseling surrogates of patients with SE, should the level of consciousness before treatment alone be considered a reliable predictor of in-hospital mortality?

#### Description of the Predictor

SE may be associated with a decreased level of consciousness, ranging from confusion to coma. The degree of impairment of consciousness is typically included in the definition of the SE subtype. Level of consciousness impairment may be measured using a variety of scales, although the Glasgow Coma Scale is most common. Level of consciousness was also categorized as awake/confused vs stuporous/comatose in some studies.

#### Recommendation

When counseling surrogates of patients with SE, we suggest the level of consciousness before treatment alone not be considered a reliable predictor of in-hospital mortality (weak recommendation; very-low-quality evidence).

#### Rationale

The body of evidence was downgraded for risk of bias in the QUIPS domains of study confounding and statistical analysis [13, 50, 51, 57, 64]. Inconsistency and imprecision were present. The level of consciousness may be influenced by multiple variables. Although subtypes of SE associated with severely decreased consciousness, such as nonconvulsive SE with coma, are more commonly associated with worse prognosis, a substantial subset of these patients may have an excellent recovery. In addition, level of consciousness was assessed at variable time points during an SE episode in the literature. Uncertainty about timing of assessment—before or after treatment with drugs such as benzodiazepines, which may depress the level of consciousness—further increases the risk of bias with this predictor.

#### Question 7

When counseling surrogates of patients with SE, should SE duration alone be considered a reliable predictor of in-hospital mortality?

#### Description of the Predictor

SE is defined either as a series of seizures without interictal clinical recovery or as a prolonged seizure not likely to be terminated by endogenous seizure-suppressing mechanisms [1]. The time span used in this definition has recently been changed to five or ten minutes, depending on the type of SE. Although the minimum seizure duration required to meet criteria for SE is only a few minutes, RSE and SRSE may persist for weeks or months, illustrating the wide spectrum of SE duration.

#### Recommendation

When counseling surrogates of patients with SE, we suggest SE duration alone not be considered a reliable predictor of in-hospital mortality (weak recommendation; very-low-quality evidence).

#### Rationale

The body of evidence was downgraded for risk of bias in the QUIPS domains of study confounding and statistical analysis. Inconsistency and imprecision were present. SE duration, as a clinical variable, is difficult to measure. The onset of SE may not be witnessed. Termination of the overt clinical manifestations of SE may be followed by ongoing electrographic seizures [66]. Termination of electrographic seizures may be challenging to determine when SE has persisted for a long time and EEG interpretation becomes increasingly difficult [1, 27]. There is evidence from animal models that seizures lead to brain damage, both in the area where the seizure activity is located and in distant cerebral structures [67]. It is unclear, however, whether these findings also apply to humans, although signals of harm exist in magnetic resonance imaging studies [68]. It appears logical that a very brief SE episode in which the first lines of treatment abort seizure activity carries a more favorable prognosis, especially quoad vitam. A rapid response to treatment may suggest a more benign underlying pathology. Also, the lower risk of complications in short SE episodes may decrease the risk of death. In episodes that do not respond to the first lines of treatment, the prognostic value of SE duration is more uncertain [46, 47, 51, 64]. Because death in patients with extremely long SE episodes frequently occurs following WLST, a high risk of bias from the self-fulfilling prophecy exists. Recovery to an excellent outcome or the patient’s baseline has been reported in several patients despite very long duration of SE [38]. SE duration may be one component of a clinical prediction model that includes variables such as etiology and diagnostic evaluation and, particularly, imaging.

#### Question 8

When counseling surrogates of patients with SE, should development of RSE alone be considered a reliable predictor of in-hospital mortality?

#### Description of the Predictor

According to the most commonly used definition, SE that proves intractable to at least the first two lines of adequately dosed ASM is termed RSE [10]. Because the probability of aborting seizures with consecutive ASMs is inversely proportional to the number of prior treatment failures, development of RSE typically indicates severity of disease and may require induction of a therapeutic coma [7].

#### Recommendation

When counseling surrogates of patients with SE, we suggest development of RSE alone not be considered a reliable predictor of in-hospital mortality (weak recommendation; very-low-quality evidence).

#### Rationale

The body of evidence was downgraded for risk of bias in the QUIPS domains of study participation and statistical analysis. Risk of bias was also present as a result of variable definitions of RSE and uncertainty about adequate dosing of ASM. Although the majority of studies pointed toward an increased risk of death with RSE [38, 46, 50], inconsistency and imprecision were both present [46, 69]. Given the large spectrum of diagnoses, presentations, and outcomes encompassed by the definition of RSE, this predictor was not considered reliable.

#### Question 9

When counseling surrogates of patients with SE, should development of SRSE/need for treatment with anesthetics alone be considered a reliable predictor of in-hospital mortality?

#### Description of the Predictor

Induction of therapeutic coma with anesthetic agents may be required to terminate seizure activity, representing the third stage of the escalating cascade of SE treatments advocated by most neurological societies worldwide. Anesthetics are often used after several intravenously administered nonanesthetic ASMs have been unsuccessful and are considered the most aggressive form of SE treatment. However, the use of anesthetics may be necessary for reasons other than termination of seizures, particularly to protect the airway in patients with SE who are comatose because of the underlying disease. Seizures that last longer than 24 h with continuous infusion of anesthetics or recur after termination of anesthetic treatment define SRSE.

#### Recommendation

When counseling surrogates of patients with SE, we suggest development of SRSE/need for treatment with anesthetics alone not be considered a reliable predictor of in-hospital mortality (weak recommendation; very-low-quality evidence).

#### Rationale

The body of evidence was downgraded for the risk of bias in the QUIPS domains study confounding and statistical analysis. The majority of studies showed an increased risk of in-hospital death in patients with SE requiring anesthetics [52], but CIs were often wide [46, 54, 60], indicating imprecision. Furthermore, one study yielded inconsistent results, depending on whether SE started inside or outside a hospital [46]. Because WLST is a major determinant of mortality, particularly in SRSE, studies of the prognostic value of anesthetic medication inherently carry the risk of a self-fulfilling prophecy.

## Recommendations: Clinical Prediction Models

### Outcome: In-Hospital Mortality

#### Question 1

When counseling surrogates of patients with SE, should the STESS be considered a reliable predictor of in-hospital mortality?

#### Description of the Predictor

The STESS is a prognostic score designed for the prediction of in-hospital mortality in patients with SE, ranging from 0 to 6 points. Points are assigned in the following categories: level of consciousness (awake/confused = 0 points; stuporous/comatose = 1 point), worst seizure type (simple partial, complex partial, absence = 0 points; generalized convulsive = 1 point; nonconvulsive in coma = 2 points), age (< 65 years = 0 points; ≥ 65 years = 2 points), and history of previous seizures (yes = 0 points; no = 1 point) [13]. A cutoff of 3 or more points may discriminate SE hospital survivors from nonsurvivors [49].

#### Recommendation

When counseling surrogates of patients with SE, we suggest the STESS not be considered a reliable predictor of in-hospital mortality (weak recommendation; low-quality evidence).

#### Rationale

The body of evidence was downgraded for risk of bias primarily in the PROBAST statistical analysis domain. Furthermore, concern about applicability was present because of variability in participants and study settings. Discrimination was reported as moderate to acceptable, with AUCs ranging from 0.6 to 0.8 [13, 56, 65, 70–80]. Most studies did not assess calibration. There was uncertainty in the literature regarding the optimal cutoff to discriminate survivors from nonsurvivors (3 vs. 4 points on the STESS) [47]. The positive predictive value for in-hospital mortality ranged from 20–40%, and negative predictive value was 85–95% (mostly with a cutoff STESS of 3) [49, 65, 72, 75, 76, 80, 81]. The STESS was not considered reliable, given the uncertainties regarding the optimal cutoff for distinguishing SE survivors from nonsurvivors, the reported low positive predictive value for mortality, and the moderate AUCs. The high negative predictive values may reflect overall low SE mortality rates rather than excellent performance of the score.

#### Question 2

When counseling surrogates of patients with SE, should the mSTESS be considered a reliable predictor of in-hospital mortality?

#### Description of the Predictor

The mSTESS is based on the STESS but uses a different cutoff for assigning points according to age (≥ 70 instead of 65 years) and includes premorbid clinical function as assessed by the mRS (mRS 0 = 0 points, mRS 1–3 = 1 point, mRS ≥ 4 = 2 points), resulting in a score ranging from 0 to 8 [74].

#### Recommendation

When counseling surrogates of patients with SE, we suggest the mSTESS not be considered a reliable predictor of in-hospital mortality (weak recommendation; low-quality evidence).

#### Rationale

The body of evidence was weaker overall compared with the STESS and was downgraded for risk of bias primarily in the PROBAST analysis domain. There were also concerns about applicability because of variability in participants and study settings. The majority of studies did not report an assessment of calibration. The discriminatory capacity of the score, as measured by AUCs in validation studies, varied between ~ 0.6 and 0.75 and appeared to be comparable to the STESS in this regard. Similarly, the positive and negative predictive values were similar to those reported for the STESS [80, 82–84]. The fact that the inclusion of premorbid function does not lead to improved prediction of in-hospital mortality in SE cohorts reflects the considerations expressed above in the sections on age and burden of comorbidities. Premorbid function does not necessarily correlate with severity of the acute illness or underlying etiology, which may be the most important determinant of survival in many patients.

#### Question 3

When counseling surrogates of patients with SE, should the EMSE be considered a reliable predictor of in-hospital mortality?

#### Description of the Predictor

The EMSE was developed using epidemiological data from existing studies. The EMSE–EACE model is a complex score encompassing numerous etiologies (E) with increasing EMSE points according to severity, age (A) stratified in ten-year intervals, comorbidities (C, based on the CCI), and EEG findings (E).

#### Recommendation

When counseling surrogates of patients with SE, we suggest the EMSE not be considered a reliable predictor of in-hospital mortality (weak recommendation; moderate-quality evidence).

#### Rationale

The body of evidence was downgraded for risk of bias primarily in the PROBAST analysis domain. Concern about applicability was present because of variability in participants and study settings. Most studies did not report on calibration. The high discrimination (AUC 0.9) reported in the initial study [65] could not be reproduced in validation studies, that reported AUCs between 0.69 and 0.86 [70, 71, 78, 79]. Notably, several studies investigated outcomes beyond hospital mortality, including mortality at three months after SE onset, and mortality at “end of study.” Variations of the EMSE include the EMSE–EAC model, which excludes the EEG domain [70, 73, 85], and the EAMSE-EAL, which includes variables the creators of EMSE-EACE tested and discarded [73, 80]. There is insufficient evidence to support the use of the EMSE in counseling surrogates of individual patients with SE regarding in-hospital mortality, although the score yielded promising results in specific SE cohorts and appears to outperform the STESS score in predicting in-hospital mortality. Similar to STESS, the optimal cutoff for discrimination of survivors from nonsurvivors has not been established.

A summary of all recommendations including good practice statements is in Table 3.

**Table 3:** Summary of recommendations: neuroprognostication in status epilepticus: good practice statements and predictors of in-hospital mortality

Good practice statements
1. We recommend that clinicians establish with patient surrogates the most appropriate long-term goals of care in individual patients with status epilepticus. Such goals, which may range from control of seizures to a return to functional independence, should serve as the basis for prognostication in an individual patient. (strong recommendation, evidence cannot be graded) 2. Surrogates of patients with refractory or super-refractory status epilepticus should be counseled that treatment measures such as prolonged sedative infusions and long-acting antiseizure medications may result in prolonged coma and dependence on life sustaining measures such as an artificial airway and artificial enteral nutrition for a variable duration ranging from days to months. Surrogates should also be counseled that medical complications related to critical illness, hospital acquired infections, and prolonged restriction of mobility are common in such patients. (strong recommendation, evidence cannot be graded) 3. Surrogates of patients with refractory or super-refractory status epilepticus should be counseled that a long-term recovery that reaches or approximates the patient’s baseline level of functioning is feasible. Factors such as underlying acute brain injury, comorbidities, nosocomial infections and sequelae of critical illness may, however, limit functional recovery. (strong recommendation, evidence cannot be graded) 4. We recommend that in patients with status epilepticus and an established underlying etiology, validated predictors of outcome specific to the underlying disease serve as a basis for neuroprognostication. (strong recommendation, evidence cannot be graded) 5. We recommend that neuroprognostication, where feasible, should be performed in the absence of potential confounders of the neurological examination, such as residual sedation, a toxic-metabolic encephalopathy or prolonged post-ictal encephalopathy. (strong recommendation, evidence cannot be graded) 6. We recommend that in patients with status epilepticus of unclear etiology neuroprognostication be deferred until an appropriate diagnostic evaluation is performed for the underling disease process. Evaluation should be timely and, where feasible, should be performed concurrently with treatment of seizures, since etiology may determine appropriate treatment and prognosis. (strong recommendation, evidence cannot be graded)
**Predictors of in-hospital mortality**
**Baseline patient characteristics**
1. When counseling family members/surrogates of patients with status epilepticus, we suggest that the **patient’s age** alone not be considered a reliable predictor of in-hospital mortality. (weak recommendation; low quality evidence) 2. When counseling family members/surrogates of patients with status epilepticus, we suggest that the **extent of comorbidities (as measured by the Charlson Comorbity Index)** alone not be considered a reliable predictor of in-hospital mortality. (weak recommendation; moderate quality evidence)
**Status epilepticus etiology**
1. When counseling family members/surrogates of patients with status epilepticus, we suggest that an **acute symptomatic or a potentially fatal etiology** alone not be considered a reliable predictor of in-hospital mortality. (weak recommendation; very low quality evidence) 2. When counseling family members/surrogates of patients with status epilepticus, we suggest that a **history of previous seizures/epilepsy** alone not be considered a reliable predictor of in-hospital mortality. (weak recommendation; very low quality evidence)
**Status epilepticus characteristics and response to treatment**
1. When counseling family members/surrogates of patients with status epilepticus, we suggest the **seizure type/semiology** alone not be considered a reliable predictor of in-hospital mortality. (weak recommendation; very low quality evidence) 2. When counseling family members/surrogates of patients with status epilepticus, we suggest** level of consciousness before treatment** alone not be considered a reliable predictor of in-hospital mortality. (weak recommendation; very low quality evidence) 3. When counseling family members/surrogates of patients with status epilepticus, we suggest **SE duration** alone not be considered a reliable predictor of in-hospital mortality. (weak recommendation; very low quality evidence) 4. When counseling family members/surrogates of patients with status epilepticus, we suggest **development of refractory status epilepticus** alone not be considered a reliable predictor of in-hospital mortality. (weak recommendation; very low quality evidence) 5. When counseling family members/surrogates of patients with status epilepticus, we suggest **development of** **super-refractory status epilepticus/need for treatment with anesthetics** alone not be considered a reliable predictor of in-hospital mortality. (weak recommendation; very low quality evidence)
**Prediction models**
1. When counseling family members/surrogates of patients with status epilepticus, we suggest the **Status Epilepticus Severity Score (STESS)** not be considered a reliable predictor of in-hospital mortality. (weak recommendation; low quality evidence) 2. When counseling surrogates of patients with SE, we suggest the **modified Status Epilepticus Severity Score (mSTESS)** not be considered a reliable predictor of in-hospital mortality. (weak recommendation; low quality evidence) 3. When counseling family members/surrogates of patients with status epilepticus, we suggest the **Epidemiology-based mortality score in status epilepticus (EMSE)** not be considered a reliable predictor of in-hospital mortality. (weak recommendation; moderate quality evidence)

## Future Directions

Although these guidelines provide broad principles of neuroprognostication in adult patients with SE, as detailed in the good practice statements, our systematic review yielded a striking lack of reliable predictors to serve as the basis of prognostication while counseling surrogates of patients who may be dependent on life-sustaining therapy. Although the systematic review focused on mortality, there is insufficient evidence for any poor outcome following SE. Although multiple variables were independent predictors of poor outcome, the body of evidence suffered several limitations, and a significant subset of patients with these variables were, nevertheless, able to achieve a good outcome. Although SE prediction models may be useful in the setting of research and quality assessment, to facilitate comparison of populations while adjusting for severity of illness, no model achieved sufficient reliability for use in the counseling of surrogates of individual patients who were critically ill and at risk for withdrawal of life support. Our review highlights the gaps and opportunities in the literature on prognostication following SE. Predicting treatment outcomes in SE is made challenging by its numerous etiologies, wide spectrum of manifestations, and variable disease course, ranging from patients easily managed with a single dose of medication to those who require weeks of neurocritical care. In addition to the complexity of the disease itself, there is significant variability in the literature regarding the assessment of baseline clinical parameters, including SE-specific variables such as etiology and semiological features. Because assessments of functional outcomes are inconsistent in the literature and few studies assess long-term cognitive and neuropsychiatric outcomes, the panel determined that it would be inappropriate to assess the value of clinical predictors or prognostic models for outcomes other than mortality. Prediction of functional, cognitive, and neuropsychiatric outcomes is critical, however, particularly in the context of counseling family members/surrogates. In addition, the development of epilepsy in patients with de novo SE may be an important patient-centered outcome.

To improve the understanding of SE prognosis, further studies in this field should adhere to the following principles: (1) Outcome assessment should be conducted at defined time points using standardized and validated tools, including longer-term outcomes at least 6 to 12 months after hospital discharge. In addition to mortality, it is critical that cognitive, functional, and neuropsychiatric outcomes are routinely assessed. (2) Consensus should be achieved regarding outcomes and critical variables in studies of SE. These variables should include semiological information (including semiological changes over time), EEG data (including development over time), and medications administered (including doses and plasma concentrations). (3) Multicenter registries with common data elements and definitions are required to achieve a sufficient sample size and allow the creation of SE subgroups reasonably comparable in etiology, semiology, baseline clinical characteristics, and response to treatment. Data from such registries will greatly improve the quality of prognostication studies. (4) Studies should use standardized definitions and nomenclature of SE etiology, semiology, refractoriness, and electrographic correlates [1, 9, 86]. Future research on prognostication following SE may also focus on biomarkers of neurological injury, such as neuron-specific enolase, which varies in SE subtypes [87]. In addition, magnetic resonance imaging may help identify structural alterations following SE that may impact prognosis [88].

## Conclusions

These guidelines are intended to provide guidance to clinicians counseling family members and surrogates of patients with SE regarding prognosis. Although broad principles of neuroprognostication were established, the systematic review did not yield any reliable predictors of poor outcome in patients with SE who are critically ill who may be dependent on life-sustaining therapy.

## Conflicts of interest

S.Mu. has received research funding from the National Institutes of Health (R21NR020231, U01NS119647, and U01NS099046) and speaking and writing honoraria from the American Academy of Neurology for topics related to neuroprognostication. She receives consultant fees for serving on the End Point Adjudication Committee for Grace Therapeutics Pharma Inc. for a clinical trial related to SAH. K.E.W. received research funding from the German NeuroIntensive Care Society (DGNI) and the B. Braun Foundation and serves as an associate editor for *Neurocritical Care*. K.M.B. has received consulting fees from Rissman Law and serves as an associate editor for *Neurocritical Care*. D.Y.H. has received research funding from the Neurocritical Care Foundation and National Institute of Neurological Disorders and Stroke (NINDS) and serves as an associate medical director for New England Donor Services. S.Ma. has received consulting fees from Marinus and speaking honorarium from the American Academy of Neurology (AAN) and Society of Critical Care Medicine (SCCM). G.V.F. has received consulting fees and speaking honoraria from the Anticoagulation Forum, AstraZeneca, and Chiesi; he is on the board of directors of the Anticoagulation Forum; and he has received advisor fees from Annexon Biosciences. T.W. has received research funding from the Else-Kroener-Fresenius-Stiftung and consulting fees and honoraria from Medtronic Navigation. The remaining authors have no conflicts to disclose related to the content of this manuscript.

## Supplementary Information

Below is the link to the electronic supplementary material.

Supplementary file1 (DOCX 79 kb)
